# The role of IL-33/ST2 axis in esophageal inflammatory diseases and cancers: implications for the immunopathogenesis and immunotherapeutic target?

**DOI:** 10.1177/17562848251344049

**Published:** 2025-06-12

**Authors:** Gaofeng Lu, Guanglin Cui

**Affiliations:** Research Group of Gastrointestinal Diseases, The Second Affiliated Hospital of Zhengzhou University, Zhengzhou, China; Faculty of Health Science, Nord University, Campus Levanger, Levanger 7600, Norway; Research Group of Gastrointestinal Diseases, The Second Affiliated Hospital of Zhengzhou University, Zhengzhou, China

**Keywords:** esophagus, IL-33, inflammation, ST2, tumorigenesis

## Abstract

Considerable scientific evidence confirms that interleukin (IL)-33 and its main receptor, suppression of tumorigenicity 2 (ST2), form a functional axis to modulate the development of esophageal inflammatory disorders, such as eosinophilic esophagitis and gastroesophageal reflux disease. Recently, studies have also revealed that the IL-33/ST2 axis is implicated in the immunopathogenesis of both esophageal adenocarcinoma and squamous cell carcinoma. In view of the importance of the IL-33/ST2 axis in the immunopathogenesis of esophageal inflammatory diseases and cancers, this review summarizes recent progress in this research field based on current published data. Moreover, the translational potential of the IL-33/ST2 axis as a promising immunotherapeutic target in esophageal inflammatory disorders and cancers was discussed.

## Background

Interleukin (IL)-33 belongs to the IL-1 cytokine family with a strong capacity to trigger the inflammatory cascade by activating nuclear factor-κB (NF-κB) signaling pathway and then increases the production of cytokines, that is, IL-1β, IL-3, IL-4, IL-5, IL-6, IL-13, and tumor necrosis factor (TNF)-α, to promote the process of inflammation in different cells/tissues.^1,2^ The biological function of IL-33 in promoting inflammation is mediated by its receptor suppression of tumorigenicity 2 (ST2) expressed in diverse target cells. Therefore, IL-33, together with its receptor ST2, forms a functional axis to participate in the modulation of inflammatory disorders.^3,4^ Since chronic inflammation has long been recognized as one of the main driving factors for cancer development,^5,6^ there is also great interest in evaluating the role of IL-33/ST2 axis in the progression of many types of cancers.^7–10^

Recently, the role of IL-33 in esophageal diseases has been studied. Increasing evidence suggests that IL-33 contributes to the induction of chronic inflammation in the esophagus, for example, elevated IL-33 levels are associated with the inflammation process in patients with eosinophilic esophagitis (EoE)^11–13^ and gastroesophageal reflux disease (GERD).^14,15^ Moreover, animal studies revealed that overexpression of IL-33 in the esophageal squamous epithelium could induce EoE-like pathological characteristics in IL-33 transgenic mice.^12,16^ Apart from the findings from EoE, recent findings suggest that IL-33 is also involved in the pathogenesis of esophageal cancers. For instance, Liu et al.^17^ reported a promoting effect of IL-33 on the growth and invasion of esophageal adenocarcinoma (EAC), and our group^18–20^ demonstrated increased expression of both IL-33 and ST2 in cancer cells, immunosuppressive cells, vascular endothelial cells (ECs), and stromal cells in human esophageal squamous cell carcinoma (ESCC).^18^ These results may suggest that IL-33 may play a potential role in both EAC and ESCC by stimulating proliferation, modulating immunosuppression, and enhancing angiogenesis.

To highlight the understanding of the role of the IL-33/ST2 axis in the immunopathogenesis of esophageal inflammatory diseases and cancer, we undertake this review to summarize the recent progress in this field and discuss its therapeutic potential in treating esophageal inflammatory diseases and cancers.

## Cellular types of the IL-33/ST2 expression in the esophagus

The analysis of IL-33/ST2 expressing cellular phenotypes may provide important histological information for understanding their biological effects and cellular targets on the human esophageal cells. Current available studies regarding IL-33/ST2 expression in human esophageal mucosa show that immunoreactivities of IL-33 and ST2 are frequently observed in numerous cellular phenotypes, including basal layer epithelial cells, immune cells, eosinophils, myofibroblasts, and microvascular ECs.^1,18–21^ In addition, ST2 is identified in mast cells, basophils, innate lymphoid cells, and eosinophils.^22^ Therefore, both IL-33 and its functional receptor ST2 are constitutively expressed in a broad range of cells in the esophageal tissues.

In the absence of proinflammatory stimuli, IL-33 is usually expressed in the nucleus.^2,23^ However, the relocation of IL-33 from the nucleus to the cytoplasm has been observed.^24^ For example, the expression of IL-33 in the cytoplasm is increased in the AGS gastric cancer cell line in response to adequate *Helicobacter pylori* stimulation.^25^ In EAC animal models, Liu et al.^17^ reported that IL-33 immunoreactivity is localized in the cytoplasm of EAC cells. While IL-33 is expressed in ESCC cells, it is predominantly expressed in the nucleus of ESCC cells.^18^ Such translocation from the nucleus to the cytoplasm may reflect that extracellular release of IL-33 synthesized in the cell nucleus must pass through the cytoplasm to the outside of the cell.^24,26^ However, the precise mechanism and significance of IL-33 translocation remain to be investigated.

Moreover, IL-33 has been shown to have a significant effect on tumor growth and progression by enhancing angiogenesis in distinct types of cancer. Studies have reported that IL-33 can potentially stimulate the proliferation and growth of vascular ECs by binding to its receptor ST2^20,23,27^ and/or activating Akt signaling to directly enhance angiogenesis.^23,28^ In addition, IL-33 could also activate stromal myofibroblasts to produce the matrix metallopeptidase (MMP)-2 and MMP-9 to promote tumor angiogenesis.^29^ Interestingly, we have found that both IL-33 and ST2 are highly expressed in microvascular ECs in the ESCC microenvironment,^18,20^ suggesting that vascular ECs are not only the target for IL-33, but also a major cellular source for IL-33.

Taken together, the identification of IL-33/ST2 expressing cells may partially explain why IL-33 has multiple biological functions by affecting diverse types of cells in the context of esophageal biology. Here, we summarize the cell types known to express IL-33/ST2 in human esophagus in Table 1.

**Table 1.** The cell types in human esophagus that have been documented to express IL-33/ST2.

Cell types	IL-33		ST2
	Nucleus (ref.)	Cytoplasm (ref.)	Transmembrane (ref.)
Normal epithelial cells	Positive^11,18,30,31^	Positive^17,32^	Positive^17,30^
ESCC cells	Positive^18^	Positive^32^	Positive^18^
EAC cells		Positive^17^	
Tregs	Positive^19^		Positive^19^
MDSCs			Positive (identified in other tissues)
Eosinophils			Positive^2^
Macrophages	Positive (our unpublished data)		Positive (our unpublished data)
Mast cells	Positive^11^		Positive^2^
Basophils			Positive^2,33^
Lamina propria cells	Positive^18^		Positive^18^
Vascular ECs	Positive^20^		Positive^20^

EAC, esophageal adenocarcinoma; EC, endothelial cells; ESCC, esophageal squamous cell carcinoma; IL-33, interleukin-33; MDSCs, myeloid-derived suppressor cells; ST2, suppression of tumorigenicity 2; Tregs, regulatory T cells.

## The role of IL-33/ST2 axis in esophageal inflammatory diseases

### Eosinophilic esophagitis

EoE is one of the most common chronic inflammatory disorders in the esophagus, mediated by food and airborne antigens. EoE is histologically defined by large numbers of eosinophils as well as mast cells, and clinically characterized by symptoms of esophageal dysfunction.^34,35^ Studies performed over the past several years have revealed that IL-33/ST2 signal in inflammatory esophageal tissues is activated in patients with EoE. Venturelli et al.^33^ reported that mice with ovalbumin percutaneously sensitized exhibited EoE-like pathological changes, including accumulated eosinophils and upregulated IL-33/ST2 expression in the esophagus as observed in human EoE. Increased expression levels of IL-33 have been observed in human EoE. For example, Judd et al.^11^ reported that the expression of IL-33 at the mRNA level was greatly increased in the esophageal tissues taken from pediatric patients with EoE. Bhardwaj et al.^13^ showed that topical beclomethasone dipropionate therapy results in a greatly decreases in eosinophilia number and IL-33 downstream cytokine (e.g., IL-3, IL-5, IL-13) levels in inflammatory esophageal tissues in a small cohort EoE patients, although the significance was not enough for them to report a significant improvement in clinical symptoms. It is generally recognized that IL-5 is one of the key activators of eosinophils.^36^ To evaluate the role of IL-33 in basal eosinophil homeostasis, Johnston et al.^37^ observed that the IL-5 expression level and mature eosinophil number were significantly increased under the stimulation of exogenous IL-33 in both the bone marrow and the periphery in wild-type and IL-33-deficient mice. They found that both IL-5 expression levels and mature eosinophil numbers were significantly increased, and blocking IL-5 signal with neutralizing anti-IL-5 monoclonal antibodies could ablate the IL-33-induced eosinophil number expansion. Thus, their findings defined a mechanism in which IL-5 upregulated by IL-33 was a key mediator in the IL-33-induced eosinophil expansion in EoE. After comparing the effect of IL-33 and IL-5 in activating eosinophils in the esophagus tissues, Angulo et al.^36^ have found that IL-33 could directly stimulate the activation of eosinophils as strongly as IL-5 did. Travers et al.^30^ further confirmed that the expression level of IL-33 was significantly increased in the basal layer of esophageal epithelial cells in the active stage and became normalized after remission in patients with EoE. Therefore, it is now widely accepted that activated IL-33 is associated with the development of EoE.

ST2 is the main functional receptor for IL-33. Uchida et al.^2^ find that increased expression of ST2 was observed in tissue eosinophils from patients with active EoE as compared with healthy controls and EoE tissues with remission. The activation of ST2 upon IL-33 stimulation causes the release of downstream cytokines IL-4, IL-5, and IL-13 from CD4 lymphocytes and group 2 innate lymphoid cells.^38,39^ Interestingly, these cytokines can increase the recruitment of eosinophils to the esophagus. For example, IL-13, as a key pathogenic cytokine in EoE and inflammation induced by IL-33, has recently been confirmed to enhance the infiltration of eosinophils into the esophageal mucosa in a STAT6-dependent and MID-1-dependent manner in TRAIL-deficient (Tnsf10(−/−)) or STAT6-deficient (STAT6(−/−)) mice.^40^ These findings imply that the effects of IL-33 on the development of EoE are via multiple mechanisms.

### Gastroesophageal reflux disease

GERD is the most common gastroesophageal disorder in the western world and affects 10%–20% of the population. Reflux of gastric acid and bile from the stomach cavity to the esophagus may cause a serious inflammation and damage the esophageal mucosa and function.^41^

Due to its strong proinflammatory capacity, the effect of IL-33/ST2 on the esophageal mucosal cells has been evaluated. For example, Shan et al.^15^ reported that nuclear IL-33 was significantly upregulated in normal human esophageal epithelial cells (HEECs) in response to interferon γ (IFN-γ) stimulation. In addition, the production of proinflammatory cytokines, for example, IL-6 IL-8, monocyte chemoattractant protein 1 (MCP-1), from normal HEECs was increased. By using the esophageal mucosa of GERD patients and in vitro stratified normal HEECs, they^14^ further showed that IL-33 is expressed in the nuclei of basal and suprabasal layers and the expression of IL-33 both at mRNA and protein levels is greatly upregulated in the erosive mucosa. In vitro, the combination of deoxycholic acid with IFN-γ can significantly improve the expression of IL-33. The above results imply a facilitating effect of IL-33 on the induction of esophageal inflammation.

Previously, Fitzgerald et al.^42^ have shown that the expression of proinflammatory cytokines IL-4 and IL-10 at mRNA transcript levels in Barrett’s esophageal (BE) tissue samples was significantly increased compared with non-inflamed and inflamed squamous esophageal tissue samples. Recently, Yosef et al.^43^ revealed that the level of IL-4 in the blood was statistically elevated in a small Egyptian cohort of refractory GERD patients as compared with GERD patients. Furthermore, the authors suggest that the level of IL-4 in the blood might be used as a discriminatory marker between GERD and refractory GERD patients.^43^

Findings from the main studies regarding the potential role of the IL-33/ST2 axis in EoE and GERD are summarized in Table 2.

**Table 2.** Main studies of the IL-33/ST2 axis in esophageal inflammatory diseases.

Studies^[ref.]^	Year	Models		Main findings
		Animal	Human	
EoE				
Travers et al.^21^	2016		Esophageal biopsies from EoE patients	Increased expression of IL-33 at mRNA and protein levels in the most basal layer in patients with active EoE.
Judd et al.^11^	2016	Mice treated with recombinant IL-33	Esophageal biopsies from pediatric EoE patients	Increased expression of IL-33 in biopsies of human EoE; mice treated with recombinant IL-33 induce EoE-like pathological features, implicating IL-33 in EoE immunopathogenesis.
Venturelli et al.^33^	2016	Mice percutaneously sensitized with OVA	Esophageal biopsies from EoE patients	Mice with OVA percutaneous sensitization result in the accumulation of eosinophils, upregulated expression of IL-33/ST2, and EoE-like features in the esophagus, which is validated in esophageal biopsies from human EoE.
Johnston et al.^37^	2016	IL-33- and ST2-deficient mice		Administration of IL-33 in wild-type and IL-33-deficient mice can significantly increase mature eosinophils in the esophagus, which can be blocked by an anti-IL-5 antibody. This finding indicates that IL-33 is necessary for homeostasis and the survival of mature eosinophils.
Travers et al.^30^	2017		Esophageal biopsies from EoE patients	Increased expression of IL-33 at the protein level is observed in undifferentiated, non-dividing esophageal epithelial cells and becomes normalized after remission.
Ishihara et al.^44^	2017		Serum from patients with EoE	The value of IL-33 in serum is undetectable in many EoE patients or control subjects.
Angulo et al.^36^	2019		Human eosinophils	IL-33 stimulates eosinophil activation.
Doyle et al.^16^	2019	iEoE33 transgenic mice		Overexpression of IL-33 in iEoE33 transgenic mice induces EoE-like pathological changes in the esophageal epithelium.
Uchida et al.^45^	2020		Esophageal biopsies from EoE patients	ST2 is overregulated in esophageal eosinophils from EoE patients. In addition, esophageal eosinophils expressed high levels of IL-5 and IL-13 in the setting of active EoE.
Marwaha et al.^46^	2022		A 12-year-old male patient with EoE	A rare and novel chromosomal duplication of the entire IL-33 gene implicates clinical features of EoE.
Uchida et al.^2^	2022		Serum and biopsies from active EoE	Esophageal eosinophils express ST2 and Th2 cytokines, e.g., IL-3, IL-4, and IL-5, in patients with EoE.
Uchida et al.^47^	2022		Human eosinophils	In response to IL-33 stimulation, human eosinophils produce high levels of Th2 cytokines IL-13 and IL-4 in vitro.
Doyle et al.^48^	2023	Mice	HEECs	Esophageal epithelial cells exposure to sodium dodecyl sulfate displays a high production of IL-33, and mice exposure to SDS shows epithelial hyperplasia and tissue eosinophilia.
Masuda et al.^12^	2024	iEoE33 transgenic mice, ST2−/−, eosinophil-deficient, and IL-13−/− mice		Overexpression of IL-33 in esophageal epithelium results in immunopathology and clinical phenotypes resembling human EoE.
GERD				
Shan et al.^14^	2015		Endoscopic esophageal biopsies from GERD patients and HEECs	Increased expression of IL-33 is observed in esophageal epithelial cells and is in the nuclei of basal and suprabasal layers. In vitro, IL-33 is upregulated in the nuclei of basal and suprabasal layers by the stimulation of IFN-γ or combined with DCA in HEECs.
Sei et al.^31^	2016		Esophageal biopsies from patients with heartburn	Upregulated expression of IL-33 in esophageal epithelium in the heartburn group is related to the symptoms.
Shan et al.^15^	2016		HEECs	HEECs treated with IFN-γ show an upregulated expression of IL-33 and increased production of various inflammatory cytokines.

DCA, deoxycholic acid; EAC, esophageal adenocarcinoma; EC, endothelial cells; EoE, eosinophilic esophagitis; ESCC, esophageal squamous cell carcinoma; GERD, gastroesophageal reflux disease; HEECs, human esophageal epithelial cells; IFN-γ, interferon γ; IL-33, interleukin-33; MDSCs, myeloid-derived suppressor cells; OVA, ovalbumin; ST2, suppression of tumorigenicity 2.

## The role of IL-33/ST2 axis in esophageal cancers

Esophageal cancer is a common malignant disease with a poor prognosis and high death rate worldwide.^49^ The incidences of esophageal cancers vary across different geographic locations according to their sub-histological types.^49^ Multiple factors, for example, smoking, GERD, BE, high body mass index, and obesity (possibly related to the development of inflammation), a diet low in fruits and vegetables, have been associated with the increased risk for the development of esophageal cancers.^50^

Growing evidence has suggested that chronic inflammation is one of the possible promoting factors for esophageal cancer development, particularly EAC,^51,52^ IL-33, as a proinflammatory cytokine, may play an important role in the induction of inflammation in esophagus,^14,48^ and is involved in esophageal cancer initiation, invasion, and metastasis.^8^ Liu et al.^17^ report that the population of IL-33-positive cells in human EAC tumor tissues (*n* = 63) is higher than that in adjacent control tissues. In the animal model, the expression level of IL-33 mRNA is increased in rats with EAC as compared with the control rats. Data from in vitro experiments show that IL-33 stimulates cell proliferation by enhancing the release of IL-6 in EAC cell lines (OE19 and OE33). BE has been widely recognized as a premalignant lesion that can develop into EAC and published literatures have demonstrated that inflammation can significantly increase the risk and promote the establishment of BE from GERD,^53,54^ in which proinflammatory cytokines, such as IL-6,^55^ IL-8,^56^ IL-17,^57^ and TNF-α,^58^ may play a central role^53,59^ in promoting the progression of BE to EAC.^52,60^ Zhong et al.^61^ reported that the expression level of IL-4 in the tissues is increased in BE as compared with GERD and control, indicating an early upregulation of IL-4 in the premalignant stage. In Liu’s study,^17^ they report that the expression level of IL-33 is increased from low-grade to high-grade dysplasia to EAC, suggesting a gradual activation of IL-33 during the transformation of a premalignant lesion to EAC. Their results obtained from in vitro experiments confirm that the stimulation effect of IL-33 on EAC cell proliferation is mediated by ST2.^17^ Therefore, there is increasing evidence that supports the notion of IL-33 as a contributing factor for the development of inflammation and the neoplastic transformation of EAC.

The potential effect of IL-33 on ESCC has also been studied. We have conducted several clinical studies to evaluate the potential role of the IL-33/ST2 axis in human ESCC.^18–20^ Our results show that both IL-33 and ST2 are highly expressed in human ESCC cells with a high proliferative index, implying a possible promoting effect on ESCC cells.^18^ To address the involvement of the IL-33/ST2 axis for the formation of immunosuppressive milieu in the ESCC, we further reveal that increased expression of IL-33 in human ESCC is associated with the increased population of FoxP3-positive regulatory T cells (Tregs) in the ESCC microenvironment, which may contribute to the formation of a immunosuppressive milieu in the ESCC.^19^ Yue et al.^32^ have also confirmed that overexpressed IL-33 in human ESCC tissues is associated with an increased population of Tregs. They have further identified that the promoting effect of IL-33 on ESCC progression is through the upregulated expression of chemokine (C–C motif) ligand 2 (CCL-2) in the ESCC microenvironment. In vivo experiments reveal that IL-33 regulates the expression of CCL-2 through transforming growth factor-β in increased Tregs. Animal studies show that knockdown of IL-33 can remarkably decrease the development of human ESCC xenografts in BALB/c nude mice.^32^ Moreover, evidence obtained from various types of cancers suggests that the promoting effect of IL-33 on cancers is through the activation of tumor stromal cells.^62–65^ In our studies, we can demonstrate that both IL-33 and ST2 are widely expressed in ESCC stromal cells and microvascular ECs,^18,20^ suggesting a participating effect of IL-33 in the modulation of tumor stroma and angiogenesis in the ESCC microenvironment. The formation of an immunosuppressive milieu favors the initiation and progression of cancers, and studies have confirmed that the shift of macrophage polarization from Th1 to Th2 phenotypes is implicated in the establishment of immunosuppression in esophageal cancer.^66^ Mai et al.^67^ have found that increased IL-33 contributes to enhanced infiltration of M2-type macrophages into ESCC tissues by activating ornithine decarboxylase (a key enzyme that catalyzes the synthesis of polyamines). In addition, myeloid-derived suppressor cells (MDSCs) represent a heterogeneous population of immunosuppressive cells and play a crucial role in the formation of immunosuppression and promote the progression of cancers, including esophageal cancer.^68^ Recently, studies have revealed that IL-13, as a key mediator for IL-33, can significantly activate the expansion of MDSCs and Tregs in patients with esophageal cancer.^69^ Thus, current primary results suggest that IL-33 and its mediators might also contribute to the architecture of immunosuppression and angiogenesis in ESCC.

It is worth stating that the role of the IL-33/ST2 axis in different esophageal inflammatory diseases varies. In EoE, the activation of IL-33 has been well documented and significantly contributes to the development of esophageal inflammation. However, several studies have revealed that the incidence of esophageal cancer has not changed in patients with EoE. For example, Uchida et al.^70^ conducted a Swedish nationwide population study to show that EoE does not increase the risk for esophageal cancer. A cross-sectional population-based study by Syed et al.^71^ even showed that EoE can reduce the risk of esophageal cancer. Moreover, two human studies showed that ESCC patients with a higher population of eosinophils in tumor tissues exhibit a better prognosis^72^ and the density of eosinophils may be used as a biomarker to predict the potential of metastasis.^73^ Most recently, Fuller et al.^74^ revealed that esophageal epithelial remodeling in response to EoE inflammation results in distinct expansion of four suprabasal populations coupled with depletion of two basal populations, which is different from that occurred in ESCC and can limit the ESCC carcinogenesis in mice. Moreover, another study has further explored the mechanisms of eosinophils against ESCC.^75^ Jacobse et al.^75^ found that the protective effect of eosinophils is through enhanced reactive oxygen species production and decreased expression of IL-17 in mice. Therefore, a direct causation between IL-33 activation and ESCC development is still lacking. Most likely, the activated IL-33 signal in the ESCC might be an epiphenomenon that contributes to the tumor-derived esophageal inflammation and tumor angiogenesis, and immune suppression.

For GERD, low pH gastric acid reflux from the stomach to the esophagus directly damages the esophageal mucosa and structures. IL-33, as an early alarm element, will be inevitably activated, and a high amount of IL-33 will be released in response to such “dangerous” signal. Such activation is secondary to the damage in the esophageal mucosa cells caused by gastric acid. For different types of esophageal cancers, the role of IL-33/ST2 may also be different. EAC has been suggested as a kind of cancer closely associated with the developed BE (a premalignant lesion for EAC). Studies have shown that chronic inflammation may significantly increase the risk of BE development in the esophagus, and IL-33 contributes to the development of esophageal inflammation; it is reasonable to speculate that IL-33 may play a key role in the initiation of EAC.

Data from the known studies regarding the role of the IL-33/ST2 axis in esophageal cancers are summarized in Table 3.

**Table 3.** Main studies of the IL-33/ST2 axis in esophageal cancers.

Studies	Year	Models			Main findings
		Animals	Human	Cancer cell line	
ESCC					
Cui et al.^18^	2018		+		Elevated expression of IL-33 and ST2 in ESCC, stromal, and vascular ECs.
Cui et al.^19^	2019		+		ST2 is expressed in Tregs, and the increased expression of IL-33 is associated with the accumulation of Tregs in the ESCC microenvironment.
Yue et al.^32^	2020	Tumor xenograft in BALB/c nude mice	+	ESCC KYSE-450 and ECA-109	Elevated IL-33 levels in human ESCC tissues. In vitro studies showed that IL-33 promotes metastasis and invasion capacity in esophageal cancer cells. Animal studies confirmed the above findings.
Mai et al.^67^		Tumor xenograft in mice.		ESCC ECA109	IL-33 enhances the polarization of macrophages toward the M2 phenotype and contributes to the formation of immunosuppression
Liu et al.^20^	2023		+		IL-33 is greatly expressed in microvascular ECs and is associated with tumor angiogenesis
EAC					
Liu et al.^17^	2022	Rat EAC	EAC	EAC OE19 and OE33	Increased expression of IL-33 in both human and rat EAC tissues, which is associated with pathological stages; in vitro experiments, IL-33 stimulates the proliferation, migration, invasion, and EMT in OE19 and OE33 cell lines.

EAC, esophageal adenocarcinoma; EC, endothelial cells; EMT epithelial–mesenchymal transition; ESCC, esophageal squamous cell carcinoma; IL-33, interleukin-33; ST2, suppression of tumorigenicity 2; Tregs, regulatory T cells.

**Figure 1.**
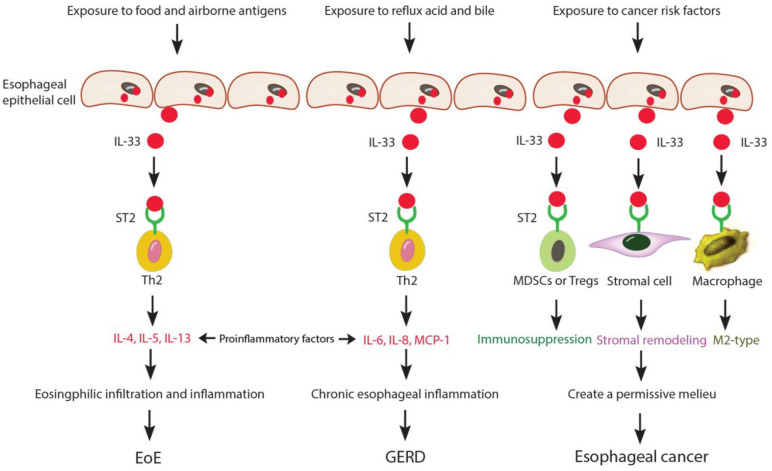
Schematic representation of postulated mechanisms for the role of IL-33 in the process of esophageal inflammation and diseases. Under the stimulation of different pathogens, the release of IL-33 from esophageal epithelial cells is significantly increased, and then affects the IL-33 receptor ST2 expressed in target cells such as Th2 cells or immunosuppressive cells (MDSCs and Tregs) or stromal cells, or macrophages. In the development of EoE, IL-33 can stimulate Th2 cells to further release downstream cytokines, for example, IL-4, IL-5, and IL-13, to promote the development of inflammation and the recruitment of eosinophils in the esophageal mucosa to induce EoE. In the development of GERD, mucosal damage induce-IL-33 stimulates Th2 cells to increase the release of IL-6, IL-8, and MCP-1 to enhance esophageal inflammation and GERD. In esophageal cancer, cancer risk factor-induced IL-33 can act on immunosuppressive cells (MDSCs and Tregs) to cause an immunosuppressive milieu, stromal cells to remodel the tumor stroma, and macrophages to cause an M2-type differentiation. All these changes contribute to the creation of a permissive microenvironment that might facilitate the progression of esophageal cancer. EoE, eosinophilic esophagitis; GERD, gastroesophageal reflux disease; IL-33, interleukin-33; MCP-1, monocyte chemoattractant protein 1; MDSCs, myeloid-derived suppressor cells; ST2, suppression of tumorigenicity 2.

Collectively, current evidence suggests that an activated IL-33/ST2 axis might have a potential effect on the development of esophageal inflammation in EoE, GERD, and esophageal cancer by multiple mechanisms. Here, we made a schematic representation to summarize the postulated mechanisms in these diseases (refer to Figure 1).

## Translational potential of the IL-33/ST2 axis as a biotherapeutic target

Extensive evidence from a variety of studies has suggested that the IL-33/ST2 axis promotes esophageal inflammation; therefore, the IL-33/ST2 axis might function as a possible biotherapeutic target for both esophageal inflammatory diseases and cancers.

### In EoE

A variety of studies confirmed that IL-4, IL-5, and IL-13 play a critical role in the induction of inflammation and EoE by IL-33.^76^ It is therefore postulated that bioagents against these cytokines may present as one of the potential therapeutics for EoE. Indeed, several clinical studies have evaluated the biotherapeutic efficacy of monoclonal antibodies specifically targeting IL-4, IL-5, and IL-13 signals in both children and adolescents with EoE.^77–79^ Results showed that targeting these downstream cytokines of IL-33 could significantly reduce the infiltration of eosinophils in the esophageal mucosa, alter histopathologic features, and improve clinical manifestations,^77–79^ suggesting that EoE patients might benefit from these targeting therapeutic strategies. Likewise, Markowitz et al.^80^ analyzed the long-term safety of Reslizumab (a humanized monoclonal antibody against human IL-5) therapy in treating both children and adolescents with EoE. They reported that both children and adolescents could well tolerate the therapy and improve clinical symptoms in EoE. However, data showed that IL-5 blockades such as mepolizumab (a fully humanized anti-IL-5 monoclonal antibody) could only partially reduce tissue eosinophilia in the esophagus, but it did not reach the primary end point of histological standard (less than 5 eosinophils/high-power field) in clinical trials.^81,82^ Therefore, the usage of anti-IL-5 antibodies in treating EoE requires more examination. More recently, de Oliveira et al.^83^ performed a systematic meta-analysis to assess the treatment efficacy and safety of monoclonal antibodies specifically targeting IL-4, IL-5, and IL-13 signals in treating EoE. They found that the administration of these neutralizing monoclonal antibodies caused a significant reduction in esophageal eosinophil numbers, potent relief of histopathologic features, and disease manifestations in EoE. Such a succession of monoclonal antibodies against IL-4, IL-5, and IL-13 resulted in great interest in using IL-33/ST2 antibodies to treat EoE. Recently, two animal experiments have assessed the effect of blocking IL-33 signals on the process of EoE in IL-33-deficient and ST2-deficient mice, respectively. Judd et al.^11^ have shown that the administration of recombinant IL-33 for 1 week in wild-type mice results in very similar histological changes in the esophagus as seen in human early EoE; these histological changes were ablated in IL-13-deficient mice. Another study performed by Nicholas et al.^33^ reported that the IL-33/ST2 axis mediates the development of EoE in mice elicited by percutaneous sensitization, and deficiency of IL-33 receptor ST2 significantly inhibits the process of EoE by a decrease in eosinophilic numbers accumulated in the esophagus of ST2^−/−^(deficient) mice. However, the clinical trials of blocking IL-33/ST signal in human EoE are currently unavailable.

### In GERD

One of the key histological features of GERD is the high degree of inflammation and erosion-induced symptoms by the exposure of the esophageal mucosa to refluxed gastric acid and bile. Interestingly, studies have found that GERD and EoE share an overlap of clinical features,^84^ in which GERD may cause the development of EoE, and vice versa.^85^ Thus, it has been postulated that the IL-33/ST2 axis could be involved in the immunopathogenesis of GERD^15,17^ and targeting the IL-33/ST2 signal and relevant downstream elements may reduce the process of esophageal inflammation and symptoms in GERD.^31^

The evaluation of the efficacy of anti-IL-5 monoclonal antibody therapy in severe eosinophilic asthma has given an interesting result. Gibson et al.^86^ showed that mepolizumab therapy for 24–52 weeks could significantly reduce GERD in patients with severe asthma and comorbid conditions. Liu et al.^87^ also reported a similar finding, in which mepolizumab therapy resulted in a reduced rate of GERD and anxiety/depression symptoms in patients with severe eosinophilic asthma. Heartburn is one of the most common symptoms in patients with GERD. To address the involvement of IL-33 signaling in heartburn, Sei et al.^31^ examined the level of IL-33 and IL-6, IL-8, or MCP-1 mRNAs in patients with heartburn. They found that upregulated IL-33 expression is related to the symptoms of GERD, suggesting that the activation of IL-33 may contribute to heartburn and inflammation in the esophagus.^31^ Other studies further confirmed the role of IL-33 as an important contributor to esophageal inflammation^14^ by the release of proinflammatory cytokines from epithelial cells.^15^ Several monoclonal antibodies targeting IL-33 downstream cytokines have been developed and evaluated in treating patients with EoE,^88^ results showed a significant reduction in inflammation and improved symptoms after therapies.^83^ Therefore, targeting IL-33/ST2 signal can reduce the inflammation in the esophageal mucosa and clinical symptoms in GERD.

## Translational significance of IL-33/ST2 as an immunotherapeutic target in esophageal cancer

Recent studies have drawn attention to the biological role of the IL-33/ST2 axis in its effects on malignant tumors. It is evident that the deregulated IL-33/ST2 network affects the progression, metastasis, and the response to therapeutics in different types of cancers, including head and neck squamous cell carcinoma.^7,65,88–91^ Therefore, it has been postulated that immunotherapies against IL-33/ST2 signals by relevant neutralizing antibodies may offer a novel immunotherapeutic strategy for esophageal cancer.

Most current human studies are performed in ESCC. ESCC is the most common histological type of esophageal cancer found in Asian countries and some special regions in other countries. Currently, there are several studies describing the role of IL-33 in esophageal cancers. Studies showed that elevated levels of IL-33 correlated with the invasion, progression, and metastasis in human ESCC^32^ by enhanced recruitment of Tregs,^19^ pro-angiogenesis,^20^ and polarization of macrophages toward the M2 subtype.^67^ EAC is another common histological type of esophageal cancer seen in the Western world. One study revealed that IL-33 participates in the development of EAC through a BE-EAC sequence by enhancing the inflammation in animal models. The above evidence suggests that IL-33 is involved in the immunopathogenesis of both ESCC and EAC.

Unlike a large number of in vitro and in vivo studies that have evaluated the therapeutic efficacy of targeting IL-33/ST2 signal in colorectal, gastric, ovarian cancers, and melanoma,^7,8^ the evaluation of the therapeutic efficacy of targeting IL-33/ST2 signal in esophageal cancer (both ESCC and ECA) is still limited and needs more exploration, both in vivo and in vitro. Considering the safety and tolerance of anti-IL-33 antibody (etokimab), clinical trials have been assessed in patients with allergic disorders, for example, peanut allergy (No. NCT02920021),^92^ atopic dermatitis (No. NCT04212169),^93^ and other human diseases (NCT03614923 and REGN3500, see details in https://clinicaltrials.gov/). In addition, several clinical trials that evaluate the effect and safety of anti-ST2 antibodies in treating asthma (No. NCT02918019, NCT03207243, NCT03393806, and NCT04366349), diabetic kidney disease (No. NCT04170543) and eosinophilic asthma (No. NCT03469934), severe atopic dermatitis (No. NCT03533751), and chronic obstructive pulmonary disease (No. NCT03387852, GSK3772847) have been assessed (details refer to https://clinicaltrials.gov/). These findings may open a new option for potential treatment, because IL-33/ST2 signaling can be easily targeted pharmacologically.

Taken together, current data suggest that blocking IL-33/ST2 signal may reduce the degree of inflammation and symptoms in EoE and GERD; however, whether targeting the IL-33/ST2 axis can affect the development of EoE and GERD is still an uncertain issue, and more studies are needed. Moreover, the development and maintenance of esophageal inflammation are modulated by a set of proinflammatory cytokines such as IL-1, IL-5, IL-6, IL-13, etc. Blocking a single cytokine signal may only reduce the inflammation, but may not completely inhibit it. Regarding the therapeutic potential of targeting the IL-33/ST2 signal in esophageal cancers (both EAC and ESCC), information is extremely limited so far. One of the main reasons is probably that the conflicting results, both pro- and anti-tumor effects, have been reported in other types of cancers, such as gastric, colorectal, and breast cancer.^7,8,94^ Another probable reason is that the increased level of IL-33 is just an epiphenomenon in response to the process of esophageal carcinogenesis, particularly in the case of ESCC. Immunohistochemical studies have revealed that both IL-33 and ST2 were widely expressed by ESCC cells,^18^ which leads researchers to hypothesize that elevated IL-33 expression in the tumor microenvironment might be particularly involved in the progression stage of ESCC by enhancing immunosuppression,^19,67^ tumor angiogenesis,^20^ and metastasis,^19,32^ but might not act as a driving force to promote the initiation of ESCC. Therefore, the therapeutic efficacy of blocking the IL-33/ST2 axis in both EAC and ESCC remains to be studied in the future.

## Conclusion and future perspectives

Increasing supportive evidence suggests that the activated IL-33/ST2 axis may play a vital role in esophageal inflammatory diseases and cancers. The safety and efficacy of monoclonal antibodies that target IL-33/ST2 and relevant mediators have been clinically evaluated in some esophageal inflammatory diseases, such as EoE. However, the exact mechanisms implicating the IL-33/ST2 axis in the development of ESSC and EAC remain fully unclear. Therefore, future work needs to clarify the molecular signaling pathways involving immunopathogenesis and the exact effect of the activated IL-33/ST2 signal on the development and progression of esophageal cancers. Particularly, novel preclinical studies should be prioritized for research into the evaluation of the targeting effect of the IL-33/ST2 axis on the development and progression of esophageal cancers.

## Appendix

### Abbreviations

BE Barrett’s esophagus

CCL-2 chemokine (C–C motif) ligand 2

DCA deoxycholic acid

EAC esophageal adenocarcinoma

ECs endothelial cells

EMT epithelial–mesenchymal transition

EoE eosinophilic esophagitis

ESCC esophageal squamous cell carcinoma

GERD gastroesophageal reflux disease

HEECs human esophageal epithelial cells

IFN-γ interferon γ

IL interleukin

MCP-1 monocyte chemoattractant protein 1

MDSCs myeloid-derived suppressor cells

MMP matrix metallopeptidase

NF-κB nuclear factor-κB

OVA ovalbumin

ST2 suppression of tumorigenicity 2

TNF-α tumor necrosis factor-α

## Declarations

**Ethics approval and consent to participate:** Not applicable.

**Consent for publication:** Not applicable.

**Availability of data and materials:** Not applicable.
